# Gait Asymmetry in People With Parkinson’s Disease Is Linked to Reduced Integrity of Callosal Sensorimotor Regions

**DOI:** 10.3389/fneur.2018.00215

**Published:** 2018-04-04

**Authors:** Brett W. Fling, Carolin Curtze, Fay B. Horak

**Affiliations:** ^1^Department of Health and Exercise Science, Colorado State University, Fort Collins, CO, United States; ^2^Molecular, Cellular and Integrative Neuroscience Program, Colorado State University, Fort Collins, CO, United States; ^3^Department of Neurology, Oregon Health & Science University, Portland, OR, United States; ^4^Veterans Affairs Portland Health Care System, Portland, OR, United States

**Keywords:** MRI, diffusion-weighted imaging, gait, balance, transcallosal, mobility

## Abstract

**Background:**

Individuals with Parkinson’s disease (PD) often manifest significant temporal and spatial asymmetries of the lower extremities during gait, which significantly contribute to mobility impairments. While the neural mechanisms underlying mobility asymmetries within this population remain poorly understood, recent evidence points to altered microstructural integrity of white matter fiber tracts within the corpus callosum as potentially playing a substantial role.

**Objectives:**

The purpose of this study was to quantify spatial and temporal gait asymmetries as well as transcallosal microstructural integrity of white matter fiber tracts connecting the primary and secondary sensorimotor cortices in people with PD and age-matched control participants.

**Methods:**

Spatial and temporal gait asymmetry in the levodopa off state was assessed using an instrumented walkway. On the next day, diffusion-weighted images were collected to assess white matter microstructural integrity in transcallosal fibers connecting the homologous sensorimotor cortical regions.

**Results:**

People with PD exhibited significantly more temporal and spatial gait asymmetry than healthy control subjects. Furthermore, people with PD had significantly reduced white matter microstructural integrity of transcallosal fibers connecting homologous regions of the pre-supplementary motor and supplementary motor areas (SMAs), but not the primary motor or somatosensory cortices. Finally, reduced transcallosal fiber tract integrity of the pre-SMA and S1 was associated with greater step length asymmetry in people with PD.

**Conclusion:**

People with PD showed increased step length asymmetries and decreased microstructural integrity of callosal white matter tracts connecting the higher-order sensorimotor cortices (pre-SMA and SMA). The strong association between gait asymmetries and corpus collosum integrity, supports the hypothesis that reduced transcallosal structural connectivity is a significant mechanism underlying gait asymmetries in people with PD.

## Introduction

Impaired walking ability is common in persons with Parkinson’s disease (PD), typically manifesting as reduced gait velocity and step length, increased gait variability, and reduced automaticity (1). PD has also been associated with temporal and spatial asymmetries of the lower extremities during gait (2, 3). These lower extremity asymmetries significantly contribute to mobility impairments in neurologic populations who experience gait and balance dysfunction. Reduced coordination during gait (i.e., increased gait asymmetry) is associated with increased metabolic cost, postural instability, falls, and reduced quality of life in those living with PD or following a stroke (4–6). While significant asymmetries in lower extremity control typically arise from unilateral neurologic insult such as a stroke, spinal cord injury, or traumatic brain injury, the neural mechanisms underlying mobility asymmetries within people with PD remain poorly understood. Although sparsely investigated, recent work suggests that altered microstructural integrity of white matter fiber tracts within the corpus callosum may play(s) a role (7–9).

Transcallosal communication *via* the corpus callosum plays a key role in the production of integrated motor behavior to generate appropriate, coordinated motor responses on both sides of the body (10, 11). The primary motor cortices are connected to the contralateral muscles controlling movement and are also densely interconnected through the corpus callosum allowing for interhemispheric transfer of information. When precisely, bilaterally coordinating movements in time and space (e.g., walking or typing), activation of one limb has a cumulative, inhibitory effect on the ipsilateral motor cortex, obtained *via* interhemispheric communication (12, 13). The relationship between reduced transcallosal structural connectivity and impaired bimanual upper extremity control is clear and well studied (10, 11), but it remains unclear if these associations extend to bilateral control of the lower extremities as well. That is to say, reduced structural connectivity of the corpus callosum is common in PD (8, 14, 15), yet it remains to be tested how reduced transcallosal structure contributes to the lower limb asymmetries observed during gait and balance tasks.

The purpose of this study was to quantify spatial and temporal gait asymmetries (assessed *via* an instrumented walkway) as well as transcallosal microstructural integrity of white matter fiber tracts connecting the primary sensorimotor cortices and supplementary motor areas (SMAs) (assessed *via* diffusion-weighted imaging) in people with PD and age-matched control participants with no known neurologic conditions. Our overarching hypothesis was that those with PD would have increased spatial and temporal gait asymmetries during over-ground walking, associated with reduced sensorimotor corpus callosum structural connectivity compared with their age-matched counterparts.

## Materials and Methods

### Participant Demographics

We recruited 39 people with idiopathic PD and 20 age-matched healthy controls (Table 1). The protocol was approved by the Institutional Review Board of Oregon Health and Science University. All subjects gave written informed consent in accordance with the Declaration of Helsinki. Clinical, mobility, and neuroimaging testing was performed over the course of two test sessions, separated by less than 1 week. All participants with PD were tested in the OFF medication state, that is, after withholding their dopaminergic medication for at least 12 h.

**Table 1:** Participant demographics.

	Parkinson’s disease	HC
*n*	39	20
Age (years)	68.7 (8.0)	71.4 (8.1)
Sex (M/F)	26/13	7/13
Disease duration (years)	7.1 (5.7)	
MDS-UPDRS III	40.1 (13.6)	
PIGD	5.5 (3.5)	
Hoehn and Yahr	2.4 (0.6)	
MoCA	24.4 (4.1)	27.1 (1.9)
Levodopa equivalent dose (mg)	1,024 (75–8,680)	

### Mobility Assessment

Participants walked at preferred gait speed three times over an 8-m long instrumented walkway with an active area of 6 m × 0.6 m sampling at a frequency of 60 Hz (GAITRite, CIR System, Havertown, PA, USA). Spatial and temporal asymmetry in percent was calculated as follows:

|1−left/right|*100,

for step length and step time, respectively.

### Image Acquisition

Neuroimaging data were collected at the Oregon Health and Science University’s Advanced Imaging Research Center on a 3.0 T Siemens Magnetom Tim Trio scanner with a 12-channel head coil. Collection parameters were similar to previous research conducted by our lab (16). Briefly, a structural, high-resolution T1-weighted MP-RAGE sequence was collected (orientation = sagittal, echo time = 3.58 ms, repetition time = 2,300 ms, 256 × 256 matrix, resolution 1.0 mm × 1.0 mm × 1.1 mm). In addition, high angular resolution diffusion images were also acquired using a 72-gradient direction, whole-brain echo-planar imaging sequence (TR = 7,100 ms, TE = 112 ms, field of view = 230 mm × 230 mm, *b* value = 3,000 s/mm^2^, isotropic voxel dimensions = 2.5 mm^3^) and 10 non-diffusion-weighted images where the *b* value was equal to 0.

### Diffusion Tensor Imaging Analysis

Diffusion data were processed using the tools implemented in FSL (Version 5.0; www.fmrib.ox.ac.uk/fslwww.fmrib.ox.ac.uk/fsl). Diffusion date were first corrected for eddy current distortions and motion artifacts, then averaged to improve signal-to-noise ratio (17) and subsequently skull stripped (using FSL’s brain extraction tool). Non-diffusion-weighted images (B0) were also utilized for field map correction to reduce geometric distortions. Each participant’s fractional anisotropy (FA) image was subsequently normalized into Montreal Neurological Institute (MNI) space *via* linear registration and Fourier interpolation through the FMRIB linear image registration tool.

### Interhemispheric Callosal Tractography

**Figure 1:**
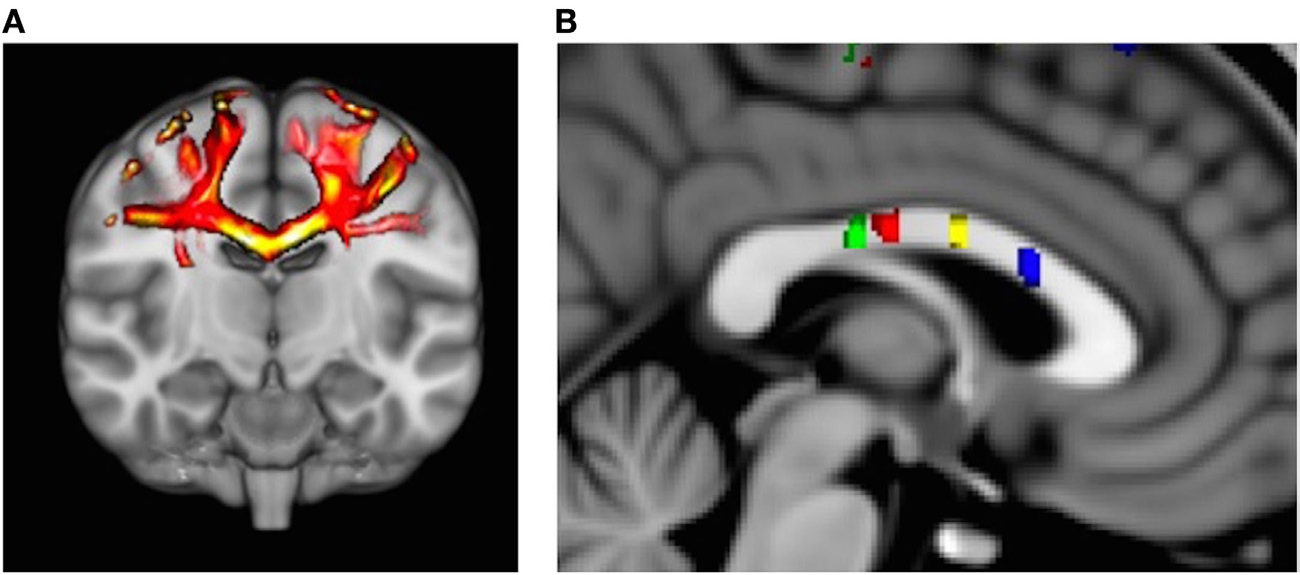
**(A)** Representative example of identified transcallosal fiber tracts connecting the homologous right and left primary motor (M1) in a participant with Parkinson’s disease. **(B)** Analysis of white matter tract microstructure was subsequently restricted to regions of interest identified on 10 mid-sagittal slices contained within the corpus callosum. Green = tracts connecting primary somatosensory; red = tracts connecting M1; yellow = tracts connecting supplementary motor area (SMA); and blue = tracts connecting pre-SMA.

Probabilistic fiber tractography to assess quantity and quality of interhemispheric structural connectivity for the body of the corpus callosum was carried out (Figure 1A). Similar to previous work (8, 18), we utilized a multiple ROI approach to provide specific fiber tract identification of callosal fibers connecting the primary and secondary sensorimotor areas. First, the Human Motor Area Template, which identifies the primary and secondary sensorimotor regions of the cortices (19), was co-registered to each individual’s MNI-normalized FA image and then used as a mask (20). The HMAT is an oft-used sensorimotor template that was identified through a meta-analysis examining functional MRI-defined cortical activity. Four sensorimotor regions were subsequently used to identify homologous, transcallosal fiber tracts connecting the SMAs and pre-SMAs, respectively, as well as the primary motor (M1) and the primary somatosensory (S1) cortices. In addition, for each interhemispheric sensorimotor fiber tract we utilized a “waypoint” ROI within the corresponding region of the body of the corpus callosum as identified by previous work (Figure 1B) (20).

For all interhemispheric sensorimotor tracts, probabilistic fiber tracking was initiated from every voxel within the binarized cortical seed HMAT ROI in each participant’s native diffusion space, was required to pass through the corresponding callosal ROI waypoint, and terminated in the contralateral hemisphere’s homologous regions ROI. We identified four distinct interhemispheric fiber tracts connecting the: (1) pre = SMA, (2) SMA, (3) M1, and (4) S1, respectively. Due to the difficulty in delineating differences between the interhemispheric connections between the ventral and dorsal premotor cortices (20), we choose to omit these ROIs from the current analysis.

As previously described (21), implicitly modeling noise in a probabilistic model allows for fiber tracking without externally added constraints such as FA threshold or fiber angle. As a result, fiber tracking in or near cortical areas, as in this study, becomes more sensitive. Utilizing a two-fiber model (22), as in this study, also improves the identification of crossing fibers. For all tractography, a large number of samples (25,000) were initiated from each voxel within the HMAT mask with the following parameters: step length of 0.5 mm, curvature threshold of 0.2. Next, the probabilistic fibers were thresholded on individual maps to include voxels with a minimum of 50% of samples [i.e., selecting all connections where >12,500 of 25,000 samples passed; a very conservative level in comparison to previous work using a threshold of 5% (20, 23)]. Finally, the identified fiber tracts were binarized and affine-transferred into MNI space and summed across participants. Analysis of tract volume and FA was calculated for all tracts identified within the four interhemispheric pathways of interest, and analysis was subsequently restricted to each participant’s 10 mid-sagittal slices (±5 slices from the mid-sagittal slice) to ensure that analysis was restricted to fiber tracts housed entirely within the corpus callosum.

### Statistical Analysis

Diffusion derived metrics were compared *via* a repeated measures analysis of variance (2 groups × 4 tracts). Larger FA values are indicative of greater directional diffusivity, which is traditionally interpreted as better white matter microstructure, e.g., denser axonal packing and higher levels of myelination (21, 22). Cohen’s *d* effect sizes to demonstrate the strength of group differences were calculated for all primary gait and fiber tract outcomes. Finally, fiber tract integrity of the four transcallosal fiber tracts were correlated with both spatial and temporal gait asymmetry in people with PD and HC, respectively. Correlations were Bonferroni-corrected for multiple comparisons and considered significant if α ≤ 0.05/2.

## Results

### Mobility Performance

**Figure 2:**
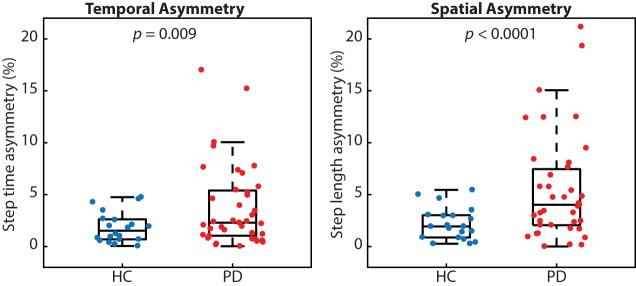
People with Parkinson’s disease (PD) exhibited significantly more temporal and spatial gait asymmetry than healthy controls (*p* = 0.009 and *p* < 0.0001, respectively).

Many people with PD walked with greater temporal and spatial gait asymmetry than age-matched healthy controls (Figure 2). While both metrics of asymmetry were greater in people with PD, we report a stronger effect size for spatial asymmetry (*d* = 0.78) as compared with temporal asymmetry (*d* = 0.59). People with PD also walked slower (*p* < 0.001; PD 1.05 ± 0.19 m/s and HC 1.28 ± 0.16 m/s) and with a shorter step length (*p* < 0.001; PD 0.55 ± 0.09 m and HC 0.66 ± 0.06 m), but similar step time (*p* < 0.48; PD 0.53 ± 0.05 s and HC 52 ± 0.04 s) compared with healthy control subjects.

### Transcallosal Fiber Tract Integrity

**Figure 3:**
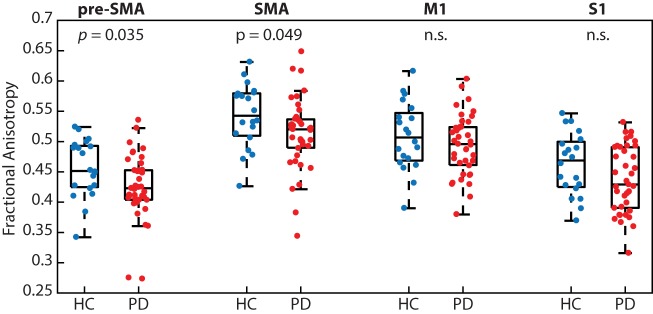
People with Parkinson’s disease (PD) had significantly reduced white matter microstructural integrity of the transcallosal fibers connecting homologous regions of the pre-supplementary motor area (SMA) and SMA, but not fibers connecting the primary motor (M1) and primary somatosensory (S1) cortices, when compared with age-matched control participants.

People with PD had significantly reduced white matter microstructural integrity (i.e., FA) of transcallosal fibers connecting homologous regions of the pre-SMA (*p* < 0.05; PD 0.42 ± 0.05 and HC 0.46 ± 0.05) and SMA (*p* < 0.05; PD 0.51 ± 0.06 and HC 0.54 ± 0.05), but not M1 (*p* = 0.34) or S1 (*p* = 0.09; Figure 3). We refer the reader to Table 2 for effect sizes comparing the strength of group differences across the four fiber tracts, which ranged from small (0.28) to medium (0.59).

**Table 2:** Cohen’s *d* effect sizes calculated for the primary gait and fiber tract sizes between groups.

	Cohen’s *d*
Temporal asymmetry	0.59
Spatial asymmetry	0.78
Pre-supplementary motor area (SMA)	0.59
SMA	0.54
Primary motor	0.28
Primary somatosensory	0.48

*A large effect (≥0.75 and <1.10); medium effect (≥0.40 and <0.75); and small effect (≥0.15 and <0.40)*.

### Associations Between Gait Asymmetry and Transcallosal Fiber Tract Integrity

**Figure 4:**
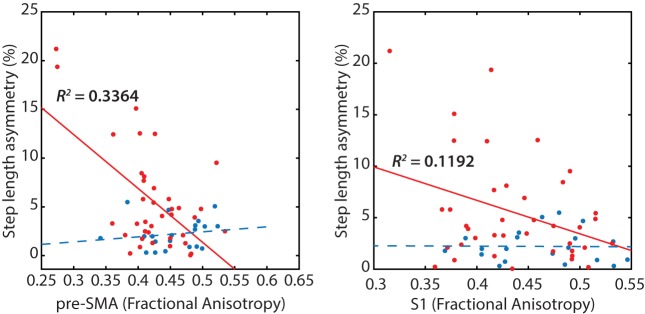
Reduced transcallosal fiber tract integrity of the pre-supplementary motor area (SMA) and primary somatosensory (S1) were associated with greater step length asymmetry in people with Parkinson’s disease, but not in healthy controls.

Poorer transcallosal tract integrity of fibers connecting the pre-SMA (*r* = −0.58; *p* < 0.001) was associated with greater step length asymmetry in people with PD, but not in healthy controls (Figure 4). In addition, FA of fiber tracts interhemispherically connecting the right and left S1 was strongly associated with spatial gait asymmetry in people with PD, although not significant when corrected for multiple comparisons (*r* = −0.34; *p* = 0.03). No significant association was observed between spatial asymmetry and tract integrity of fibers connecting either the SMA or the M1 in people with PD. In addition, no correlations were observed between temporal asymmetry and any of the four transcallosal fiber tracts (*r* < 0.19 for all) in those with PD nor were any significant correlations observed between fiber tract integrity and gait asymmetry for healthy control subjects (Table 3).

**Table 3:** Correlation between the microstructural integrity of the callosal sensorimotor regions and spatial and temporal gait asymmetry (correlations with *p* < 0.05 are highlighted in bold).

	Spatial asymmetry		Temporal asymmetry	
	Parkinson’s disease (PD)	HC	PD	HC
Pre-supplementary motor area (SMA)	**−0.5800**	0.1581	−0.0780	0.3425
SMA	−0.2699	0.2328	−0.0480	0.3744
Primary motor	−0.1367	−0.1162	0.1924	0.3813
Primary somatosensory	**−0.3452**	−0.0123	0.1923	0.0758

## Discussion

Differences in gait asymmetry between those with PD and HC were observed both spatially and temporally. Individuals with PD also had significantly reduced microstructural integrity of white matter fibers connecting the right and left pre-SMA and right and left SMA, regions responsible for higher-order motor control. Conversely, no differences in interhemispheric fiber integrity were found for those tracts connecting the right and left primary motor or somatosensory cortices. Finally, impaired neuroanatomy connecting the right and left higher-order motor planning regions of the sensorimotor cortical hemispheres (pre-SMA) and the S1 cortices resulted in a reduced capacity for spatially coordinating and controlling the legs during gait, specifically in people with PD.

### Mobility Findings

While gait is generally considered to be symmetric, subtle asymmetries do exist, even in HC (24). Findings on gait asymmetry in PD are inconsistent, while some studies have shown that gait asymmetry is increased in people with PD, especially in PD who experience freezing of gait (25, 26) others found no difference in spatial or temporal step asymmetry during over-ground walking (27). Notably, while step length asymmetry has previously been shown to be weakly correlated with disease severity (27), but not associated with asymmetry of clinical motor symptoms (25). Similar to spatial gait asymmetry (25), temporal gait asymmetry has not been found to be associated with laterality of motor symptom presentation of the disease (25, 28). Our current results demonstrate significantly increased spatial (e.g., step length) and temporal (e.g., step time) gait asymmetry in people with PD, when measured in the OFF levodopa state, when gait is most affected. While previous work has offered descriptive metrics of gait asymmetry in people with PD, the current work also provides evidence for potential neural bases underlying this altered gait pattern that appears to be independent of disease laterality as typically assessed by clinical motor assessments.

### Neuroimaging Findings

A recent meta-analysis by Atkinson-Clement et al. (15) reports consistent and significant reductions in white matter macrostructural integrity of the corpus callosum, as assessed by FA, in people with PD compared with age-matched healthy control subjects (15). While the individual contributions of axonal density and myelination to FA are not fully understood, recent work indicates that axonal membranes likely play the primary role, whereas myelination can modulate the degree of anisotropy (29). As an example, anisotropy is reduced in demyelinating disease [multiple sclerosis (30)] and in conditions of premyelination [children (31)]. While several studies have focused on the anterior portions of the callosum (i.e., the genu) and its relation to cognitive decline in those with PD (32–34), there is a small, but growing body of literature indicating associations between callosal integrity and locomotor control in PD and the elderly (8, 35).

Interhemispheric communication *via* the corpus callosum is a well-known contributor to coordinated bimanual upper extremity control in healthy (10, 36, 37) and neurodegenerative (30, 38) populations. Specifically, intact transcallosal structure has been shown to prevent interference between the two hands, particularly during bimanual out-of-phase actions (similar to gait) as compared with bimanual simultaneous movements (36, 39, 40). Providing further confirmation for the specific relationship between transcallosal connectivity and asymmetric bilateral control, those patients who have received a callosotomy maintain the capacity to synchronously coordinate their two hands while performing discrete, simultaneous actions (41). This finding indicates that bilateral coordination remains possible, via pathways distinct from callosal communication. The current work extends this relationship between integrity of transcallosal sensorimotor fiber tracts to bilateral, out-of-phase control of the lower extremities in a large sample of people with PD. Specifically, these results point to transcallosal connections between the pre-SMA and the S1 cortices as important transcallosal fiber tracts associated with reduced symmetric control of gait in PD. Somatosensory cortices and medial motor areas like the pre-SMA have oft been implicated as serving prominent roles during complex bilateral movements.

Gerloff and Andres (42) have previously identified a complex cortical network underlying bimanual coordination, and they highlight the importance of the bilateral primary sensorimotor cortices, along with medial motor wall areas including the cingulate motor area and the pre-SMA. There are particularly dense homotopic transcallosal connections within the pre-SMA and SMAs (43), and these higher-order motor regions substantially influence M1 activity in both hemispheres during the execution of visually paced movements (44). For example, the pre-SMA significantly inhibits the opposite hemisphere’s M1, thereby suppressing its activity. A growing body of literature investigating the effects of non-invasive brain stimulation (e.g., repetitive TMS) to reduce activity of the pre-SMA and SMA has shown that temporal pacing while bimanually tapping the fingers in an anti-phase pattern is selectively degraded, as compared with synchronously tapping (45). The authors suggest that deterioration of interhemispheric coupling due to stimulation likely reduces interhemispheric inhibition resulting in poorer motor performance on tasks requiring a higher level of interhemispheric inhibition. Similarly, our current results demonstrate that impaired neuroanatomy connecting the bilateral pre-SMAs results in a decreased ability to produce consistent steps with regards to the spatial domain in individuals with PD.

While the current literature is quite limited with regards to interhemispheric transfer of information between the primary somatosensory cortices, Geffen and colleagues (46) report that afferent feedback carries significant information regarding temporal control of movement and that it is a reduction in this sensory feedback that primarily impairs out-of-phase bilateral movement such as gait. Furthermore, recent work from Jung et al. (47) demonstrates that interhemispheric inhibition transmitted transcallosally between the bilateral somatosensory cortices is directly correlated with bimanual tactile performance, indicating that these interhemispheric sensory fiber tracts have behavioral importance for bimanual object manipulation and exploration. Structural findings within the brains of pianist’s, a group that demands exquisite bilateral control and coordination, also demonstrates structural adaptations/enlargements of the SI hand representation (48). The current results are the first we are aware of that demonstrate the importance of transcallosal somatosensory fiber tracts and the ability to output consistent spatial bilateral gait patterns (i.e., steps). Collectively, the pre-SMA and S1 both exert an inhibitory influence on the contralateral M1, particularly for tasks where each limb undergoes independent spatiotemporal profiles that must be coordinated together, like gait in this study.

A limitation of the current manuscript is the amount of gait captured, comprising only three trials over an 8-m walkway per participant. While the use of an instrumented walkway is required to accurately assess spatial asymmetry during typical gait, this approach has been shown to be reflective of multiple gait characteristics including gait speed, cadence, and step length and time variables, as used in this study (49).

## Conclusion

People with PD showed greater temporal and spatial gait asymmetry between the two legs along with decreased microstructural integrity of callosal white matter tracts connecting the pre-SMA and SMAs. Furthermore, strong associations were observed between callosal integrity of fiber tracts connecting pre-SMA and S1 cortical regions and step length asymmetries, solely in people with PD. These results indicate that reduced transcallosal sensorimotor structural connectivity may be a significant mechanism underlying bilateral gait asymmetries in those with PD.

## Ethics Statement

All patients or their next of kin gave informed, written consent to a protocol approved by the Institutional Review Board of Oregon Health and Science University.

## Author Contributions

BF, FH, and CC all contributed to the conceptual design and interpretation of this study. BF and CC contributed to data collection and analysis.

## Conflict of Interest Statement

The authors declare that the research was conducted in the absence of any commercial or financial relationships that could be construed as a potential conflict of interest.
